# Comparison of long-term radial artery occlusion via distal vs. conventional transradial access (CONDITION): a randomized controlled trial

**DOI:** 10.1186/s12916-024-03281-7

**Published:** 2024-02-08

**Authors:** Tao Chen, Lamei Li, Feng Li, Wei Lu, Ganwei Shi, Wenhua Li, Anni Yang, Hui Huang, Jianqiang Xiao, Qiuwei Zhang, Jun Gu, Sheliang Xue, Liuyan Zhang, Li Li, Lingxia Xu, Rongrong Ji, Haibo Wang, Gaojun Cai

**Affiliations:** 1 Department of Cardiology, Wujin Hospital Affiliated With Jiangsu University, The Wujin Clinical College of Xuzhou Medical University, Changzhou, 213017 Jiangsu Province China; 2 Department of Cardiology, Jiangyin Hospital of Traditional Chinese Medicine, Wuxi, 214400 Jiangsu Province China; 3 Department of Catheter Room, Wujin Hospital Affiliated With Jiangsu University, The Wujin Clinical College of Xuzhou Medical University, Changzhou, 213017 Jiangsu Province China; 4 Peking University Clinical Research Institute, Peking University First Hospital, Beijing, 100191 China; 5 Key Laboratory of Epidemiology of Major Diseases (Peking University), Ministry of Education, 38 Xueyuan St, Haidian District, Beijing, 100191 China

**Keywords:** Distal transradial access, Transradial access, Radial artery occlusion, Randomized controlled trial

## Abstract

**Background:**

The distal transradial access (dTRA) has become an attractive and alternative access to the conventional transradial access (TRA) for cardiovascular interventional diagnosis and/or treatment. There was a lack of randomized clinical trials to evaluate the effect of the dTRA on the long-term radial artery occlusion (RAO).

**Methods:**

This was a prospective, randomized controlled study. The primary endpoint was the incidence of long-term RAO at 3 months after discharge. The secondary endpoints included the successful puncture rate, puncture time, and other access-related complications.

**Results:**

The incidence of long-term RAO was 0.8% (3/361) for dTRA and 3.3% (12/365) for TRA (risk ratio = 0.25, 95% confidence interval = 0.07–0.88, *P* = 0.02). The incidence of RAO at 24 h was significantly lower in the dTRA group than in the TRA group (2.5% vs. 6.7%, *P* < 0.01). The puncture success rate (96.0% vs. 98.5%, *P* = 0.03) and single puncture attempt (70.9% vs. 83.9%,* P* < 0.01) were significantly lower in the dTRA group than in the TRA group. However, the number of puncture attempts and puncture time were higher in the dTRA group. The dTRA group had a lower incidence of bleeding than the TRA group (1.5% vs. 6.0%, *P* < 0.01). There was no difference in the success rate of the procedure, total fluoroscopy time, or incidence of other access-related complications between the two groups. In the per-protocol analysis, the incidence of mEASY type ≥ II haematoma was significantly lower in the dTRA group, which was consistent with that in the as-treated analysis.

**Conclusions:**

The dTRA significantly reduced the incidence of long-term RAO, bleeding or haematoma.

**Trial registration:**

ClinicalTrials.gov identifer: NCT05253820.

**Supplementary Information:**

The online version contains supplementary material available at 10.1186/s12916-024-03281-7.

## Background

In recent years, the distal transradial aceess (dTRA) has emerged as an attractive and alternative access to the conventional transradial access (TRA) for cardiovascular interventional diagnosis and/or treatment [1–3]. Compared with other approaches, cardiovascular catheterization via the dTRA not only increases patient comfort but also significantly shortens the time of compression haemostasis and reduces the incidence of vascular access-related complications [4–6]. Thus, a significant reduction in the incidence of radial artery occlusion (RAO) is an important reason for interventional doctors to choose the dTRA in cardiovascular interventions. Despite partially spontaneous recanalization, RAO is a common complication of cardiovascular interventions via the TRA, with an incidence ranging from 0.8 to 38% [7]. Although most patients with RAO lack hand ischaemia symptoms, the reuse of the radial approach is limited. Most studies have found that the incidence of early RAO is significantly lower with the dTRA than with the TRA [8, 9]. However, the DISCO RADIAL study showed that there was no significant difference in the rate of early RAO between the two groups after taking effective measures to prevent RAO [10].

Most primary endpoints in previously published randomized controlled trials (RCTs) were short-term RAO at 24 h or before discharge. The long-term outcome of RAO can better reflect the value of dTRA in the prevention of RAO. Although several studies have investigated both the early-term RAO and the late-term RAO, the follow-up times were limited. Few studies have dynamically observed changes in the rate of RAO after tranradial/distal transradial artery intervention and it is not clear when stability will be achieved. According to clinical experience, most spontaneous recanalization of RAO occurs in 3 months. In fact, there is no clear definition of “long-term” RAO follow-up in the literature. Until now, few randomized controlled studies have explored whether dTRA can reduce the incidence of long-term RAO.

Repeated ipsilateral radial artery access might aggravate damage to the radial intima, which might decrease the successful puncture rate and increase the incidence of access-related complications, including RAO [11, 12]. To date, most randomized studies comparing the effects of dTRA and TRA on the incidence of RAO have included some patients with a history of ipsilateral radial artery intervention, which might have an impact on the incidence of RAO. Therefore, the main objective of this study was to investigate whether dTRA could reduce the rate of long-term RAO in patients without a history of ipsilateral radial artery intervention.

## Methods

### Study design

The CONDITION (Comparison of Long-term Radial Artery OcclusiON in Coronary Diagnosis and/or Intervention Via DIstal vs. ConvenTIONal Transradial Access) study was a single-centre, prospective, randomized controlled trial. This study was approved by the Ethics Committee of Wujin Hospital Affiliated with Jiangsu University. Written informed consent was obtained from all of the patients. The study protocol was performed in accordance with the Declaration of Helsinki and was registered in ClinicalTrials.gov (Registration number: NCT05253820).

### Study population

**Fig. 1:**
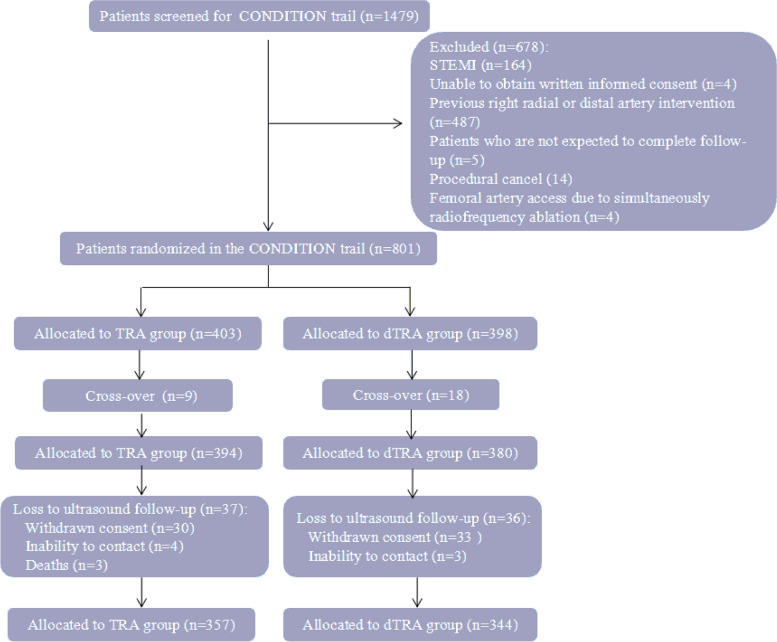
Study flowchart

Patients who were scheduled for coronary angiography and/or intervention in the cardiovascular department of our hospital between February and December 2022 were screened. The eligibility criteria included age ≥ 18 years, palpable distal and conventional radial arteries, willingness to participate in the study and a signed informed consent form. Patients with cardiogenic shock or acute ST elevated myocardial infarction were excluded. Other exclusion criteria included age ≥ 90 years, height ≥ 185 cm, previous right radial or distal artery intervention, contraindications to puncture at the puncture site, and expected loss to follow-up. Patients who had previously undergone a right radial artery intervention and then underwent coronary artery bypass grafting (CABG) with the left internal mammary artery were also enrolled in the study due to priority of left side access (Fig. 1).

### Randomization

After the patients signed the informed consent forms, they were randomized in a 1:1 ratio to the dTRA or TRA group. According to the literature, patient and procedure-related factors were found to be associated with RAO after the TRA procedure [7]. Several studies have revealed that female sex is an independently predictive factor for RAO. Compared with that in males, the diameter of dTRA in females was significantly smaller, which led to more puncture failure. In addition, the small vessel leads to a higher percentage of sheath-to-vessel mismatch and increases the incidence of RAO. Therefore, we used stratified randomization by gender to reduce the impact of gender on the study. The computer-generated randomization sequence stratified patients by sex and was performed by an independent researcher. For concealed allocation, the group allocation was stored in individually sealed envelopes that were opened in sequence by the operators prior to the beginning of the procedure.

### Study procedure

Vessel puncture was performed by five interventional cardiologists in our centre who had performed more than 500 TRA punctures and more than 50 dTRA punctures. According to the protocol of this study, the right vessel seemed to be the prior puncture site. However, in patients who had previously undergone CABG with the left internal mammary artery, the left hand was the proposed puncture site.

### dTRA puncture

Patients were supine on the digital subtraction angiography examination bed. After disinfection, the right hand was placed in a natural position on the right side of the body, and the left hand was placed in the region of the right inferior abdomen. After local infiltration anaesthesia with 1–2 ml lidocaine, puncture was performed with the Seldinger method.

The details of the dTRA puncture procedure are described elsewhere [13], but briefly, the operators palpated the artery to locate the pulse and punctured the distal radial artery with a 20-gauge trocar at a 30° angle. After a successful puncture, a 6 Fr artery sheath (RADIFOCUS INTRODUCER II, Terumo, Japan) was carefully inserted into the artery.

### TRA puncture

Unlike the puncture of the distal radial artery, the TRA puncture requires the patient’s hand to be in an external rotation position, and the puncture point was located 2–3 cm proximal to the wrist stripe. The puncture method and subsequent operation were the same as those for the distal radial artery.

After a successful puncture, 3000 U of unfractionated heparin was injected immediately, and 100 U/kg was added if a coronary intervention was needed. The arterial sheath was removed immediately after the procedure. In the TRA group, haemostasis was achieved by using a compression device (TR Band, Terumo). First, approximately 14 ml of gas was injected into the airbag until the bleeding completely stopped. Then, the gas-filled bag was slowly deflated until there was minimal bleeding again. Finally, another 2 ml of gas was added. Afterwards, 2 ml of air was released every 2 h. The compressor was completely removed after 6 h for coronary angiography (CAG) and after 10 h for percutaneous coronary intervention (PCI). In the dTRA group, an elastic bandage was used to achieve haemostasis. The folded gauze was placed on the bottom of the anatomic snuffbox (AS) and wrapped with an elastic bandage. The elastic bandage was loosened after 1 h for CAG only and after 2 h for PCI and then completely withdrawn after another 2 h.

### Ultrasound examination

The vessel was evaluated using a portable ultrasound machine with an L18-4MH frequency probe (Konica Minolta, HS1 Plus). According to the protocol, the radial artery segment from the AS to 20 cm proximal to the styloid process of the radius was evaluated. The examination included not only the internal diameter and intima thickness of the vessels but also any vascular complications such as RAO, distal radial artery occlusion (dRAO), pseudoaneurysm or arteriovenous fistula. The ultrasound was performed by an independent assessor who was not involved in the study but had extensive experience. Ultrasound examinations were performed at three time points, 1 day prior to the cardiovascular intervention and at 24 h and 3 months after the procedure.

### Study endpoints

**Fig. 2:**
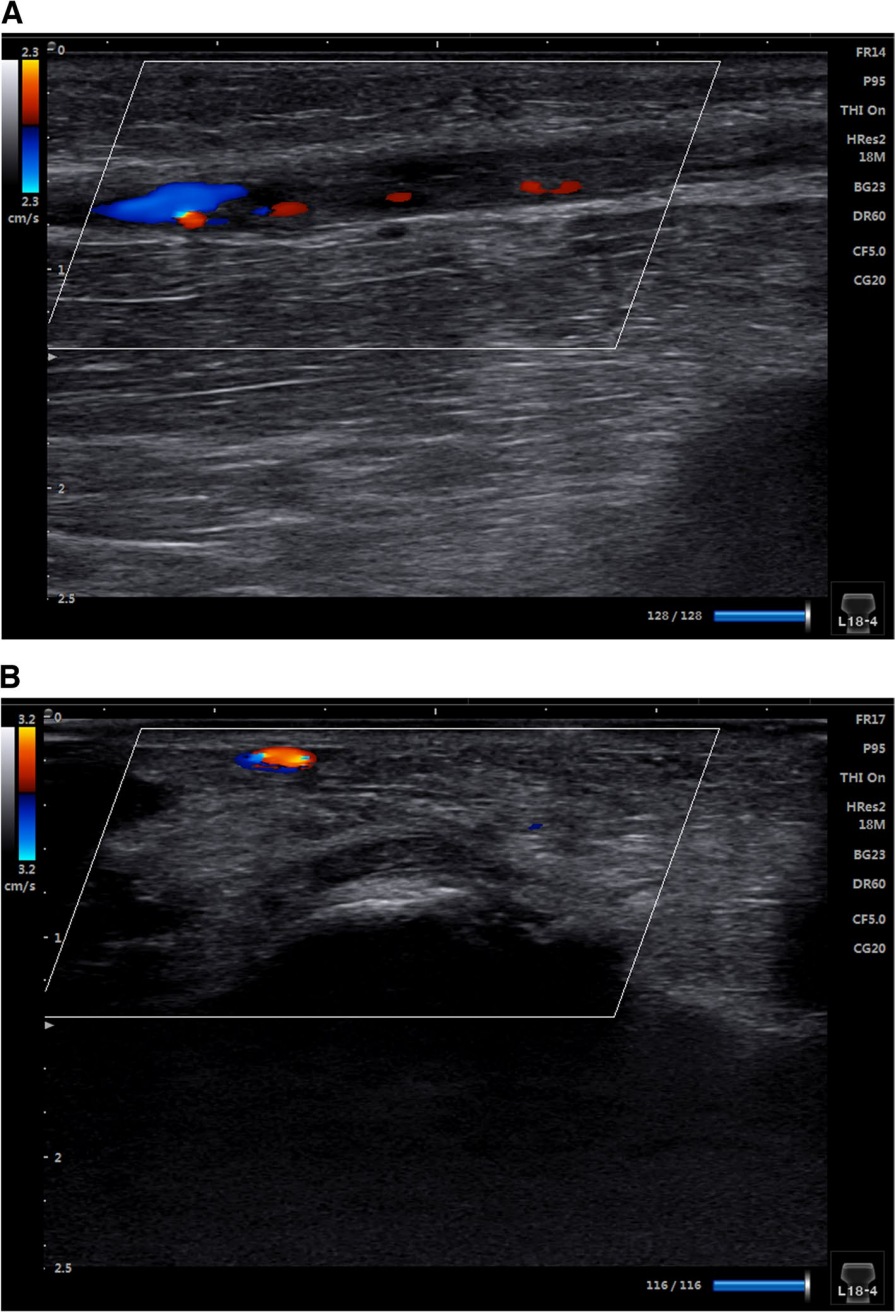
Ultrasonography of RAO and dRAO. **a** Ultrasonography of RAO. **b** Ultrasonography of dRAO

The primary endpoint was long-term RAO at the 3-month follow-up after the procedure. The criteria for RAO included thrombus, interrupted blood flow, and disappearance of the pulse spectrum of the artery, as shown by Doppler ultrasound (Fig. 2).

The secondary endpoints included the successful puncture rate, defined as complete insertion of the artery sheath; single puncture attempt success rate; puncture time, defined as the time from the end of local anaesthesia to the sheath completely implanted into the vessel; procedure time; fluoroscopy time; radiation dose; contrast volume; and access-related complications, such as finger numbness, pain during haemostasis, pseudoaneurysm and arteriovenous fistula, RAO and dRAO at 24 h. A modified version of the Early Discharge After Transradial Stenting of Coronary Arteries Study (mEASY) classification was used to evaluate the haematoma and the Bleeding Academic Research Consortium (BARC) criteria was used to evaluate the bleeding [14, 15]. Pain during haemostasis was assessed using the visual analogue scale (VAS). A score of 0 indicates no pain, 1–3 to mild pain, 4–6 to moderate pain, and 7–10 to severe pain [16].

### Statistical analysis

According to the previous literature, the incidence of RAO in the TRA group was approximately 7.7% before discharge and decreased to 5.5% after more than 1 week of follow-up [7]. The occurrence of RAO in the dTRA group was approximately 1.7%, as previously reported [17]. We assumed that the rate of recanalization was similar between the two groups and estimated that the incidence of RAO in the dTRA group was 1.2% at the 3-month follow-up. A total of 692 participants (346 in each group) should be enrolled in the study to provide 80% power to detect the 4.3% difference in the risk of primary outcome, assuming a 20% loss to follow-up rate, at a two-sided type I error of 0.05. However, approximately 800 participants were eventually enrolled in the study to achieve greater power of testing.

The primary and secondary endpoints were analysed in both intention-to-treat (ITT) and per-protocol (PP) populations, with ITT analysis as the primary analysis. The ITT population included all patients who underwent randomization and treatment. The PP population excluded patients who were not suitable for the study protocol or were lost to follow-up. In addition, an as-treated analysis was performed as a sensitivity analysis. Post hoc analysis to the primary outcome was performed by subgroup analysis stratified by age (< 60 years or ≥ 60 years), sex (male or female), body mass index (BMI) (< 24.0 kg/m^2^ or ≥ 24.0 kg/m^2^), essential hypertension (EH) (yes or no), diabetic mellitus (DM) (yes or no), atrial fibrillation (AF) (yes or no), CAG only (yes or no), radial artery diameter (< 2.3 mm or ≥ 2.3 mm). Continuous variables were expressed as the mean ± standard deviation for normally distributed data or median (interquartile range) for nonnormally distributed data, and the difference between the two groups was compared with *t* test or Wilcoxon rank test as appropriate. Categorical variables were expressed as numbers (percentages) and compared with the chi-square test or Fisher’s exact test. Risk ratios (RR) with 95% confidence intervals (CI) were used to compare the difference in the rate of RAO between the two groups. No imputation was performed for missing data. A two-tailed *P* < 0.05 was considered statistically significant. All statistical analyses were performed using SPSS 17.0 software.

## Results

Between February 2022 and December 2022, 801 of 1479 patients who were scheduled for coronary angiography and/or intervention were enrolled in the study and randomized to the dTRA group (*n* = 398) or the TRA group (*n* = 403), as shown in the flowchart (Fig. 1). The median age of the patients was 66.0 (57.5–73.0) years, and 450 (56.2%) were males. Four patients had previously undergone CABG with the left internal mammary artery, and five patients had previously undergone a cardiovascular intervention via the femoral artery.

### Baseline characteristics

Table 1 lists the baseline characteristics of the enrolled patients. The groups were comparable in terms of demographics, preoperative vital signs, medical history and laboratory findings. There was no significant difference between the two groups in terms of medication, except for aspirin (48.4% vs. 38.2%, *P* < 0.01). The ejection fraction was higher in the dTRA group than in the TRA group (63.0% vs. 62.0%, *P* < 0.01). There was no statistically significant difference in the diameter of the conventional radial artery between the two groups. However, the diameter of the distal radial artery in the TRA group was smaller than that in the dTRA group (*P* < 0.05).

**Table 1:** Baseline characteristics of the study population

Characteristic	TRA (*n* = 403)	dTRA (*n* = 398)	*P*
**Demographics**			
Age, years	67.0 (58.0–73.0) (403)	66.0 (57.0–73.0) (398)	0.13
Male, % (*n*)	56.3 (227/403)	56.0 (223/398)	0.93
Body mass index, kg/m^2^	24.5 (22.3–26.8) (403)	24.6 (22.5–26.8) (394)	0.57
**Preprocedure vital signs**			
Systolic blood pressure, mmHg	137.0 (124.0–148.0) (403)	136.0 (125.0–148.0) (398)	0.75
Diastolic blood pressure, mmHg	82.0 (76.0–90.0) (403)	82.0 (76.0–89.0) (398)	0.56
Heart rate, beat/min	72.0 (67.0–82.0) (403)	76.0 (68.0–82.0) (398)	0.17
**Medical history**			
Current smoking, % (*n*)	31.8 (128/403)	27.4 (109/398)	0.18
Hypertension, % (*n*)	62.5 (252/403)	65.8 (262/398)	0.33
Diabetes mellitus, % (*n*)	20.3 (82/403)	18.8 (75/398)	0.59
Dyslipidemia, % (*n*)	4.0 (16/403)	3.5 (14/398)	0.77
AF, % (*n*)	15.4 (62/403)	11.6 (46/398)	0.11
Previous PCI with femoral access, % (*n*)	0.5 (2/403)	0.8 (3/398)	0.64
Previous CABG with LIMA, % (*n*)	0.2 (1/403)	0.8 (3/398)	0.37
Previous stroke, % (*n*)	6.7 (27/403)	6.3 (25/398)	0.81
**Laboratory findings**			
WBC, *10^9^/l	6.3 (5.2–7.8) (402)	6.3 (5.2–7.8) (397)	0.56
Haemoglobin, g/l	138.0 (128.0–148.0) (402)	138.0 (129.0–152.0) (397)	0.31
Platelet, *10^9^/l	199.0 (166.0–240.0) (402)	204.0 (173.0–242.0) (397)	0.38
Creatinine, μmol/l	70.2 (60.0–81.9) (402)	70.4 (58.3–82.8) (396)	0.72
UA, μmol/l	339.2 (285.6–406.6) (402)	340.6 (272.9–429.8) (396)	0.91
HbA1C, %	6.1 (5.7–6.6) (378)	6.0 (5.7–6.7) (379)	0.19
TC, mmol/l	4.4 (3.5–5.1) (401)	4.5 (3.7–5.2) (398)	0.09
TG, mmol/l	1.4 (1.0–2.1) (401)	1.5 (1.1–2.22) (398)	0.06
LDL–C, mmol/l	2.4 (1.8–2.9) (401)	2.4 (1.9–2.9) (398)	0.19
APTT, s	26.9 (25.6–28.6) (395)	26.7 (25.1–28.2) (388)	0.06
D-Dimer, mg/l	0.3 (0.2–0.5) (371)	0.3 (0.2–0.5) (374)	0.46
**Echocardiography**			
LVSD, mm	32 (29–35) (379)	31 (28–33) (368)	0.01
LVED, mm	48 (45–51) (379)	48 (45–51) (368)	0.06
EF, % (*n*)	62 (57–65) (379)	63 (59–66) (368)	< 0.01
**Medication**			
Aspirin, % (*n*)	48.4 (195/403)	38.2 (152/398)	< 0.01
Indobufen, % (*n*)	4.7 (19/403)	6.3 (25/398)	0.33
Clopidogrel, % (*n*)	36.2 (146/403)	34.7 (138/398)	0.65
Ticagrelor, % (*n*)	13.2 (53/403)	10.3 (41/398)	0.21
Statin, % (*n*)	88.3 (356/403)	84.7 (337/398)	0.13
Oral anticoagulation, % (*n*)	15.6 (63/403)	11.6 (46/398)	0.09
**Diameter of vessels**			
Conventional radial artery, mm	2.3 (2.0–2.6) (403)	2.3 (2.0–2.7) (398)	0.42
Distal radial artery, mm	2.0 (1.7–2.3) (403)	2.0 (1.8–2.3) (398)	0.01

Values are % (*n*/*N*) or median (IQR) (*n*)

*TRA*, Transradial approach, *dTRA* distal transradial access, *AF* Atrial fibrillation, *PCI* Percutaneous coronary intervention, *LIMA* Left internal mammary artery, *CABG* Coronary artery bypass grafting, *WBC* White blood cell, *UA* Uric acid, *TC*, Total cholesterol, *TG* Triglyceride, *APTT* Activated partial thromboplastin time, *LVSD* Left ventricular systolic diameter, *LVED* Left ventricular end-diastolic diameter, *EF* Ejection fraction

### Procedure characteristics

The procedure characteristics are shown in Table 2. The right approach was first accepted in most cases in both groups (99.8% vs. 99.2%, *P* = 0.31). The proportion of PCI in the dTRA group was lower than that in the TRA group (22.9% vs. 29.8%, *P* = 0.03). Among PCI patients, there was no difference between the two groups in the proportion of multivessel disease or the number of implanted stents and drug balloons. The proportions of coronary artery disease (54.6% vs. 50.8%, *P* = 0.28) and acute coronary syndrome (23.1% vs. 18.3%, *P* = 0.10) were similar in the two groups. The sheath crossover from 6 to 7 Fr occurred in four cases in the TRA group. To achieve successful puncture, the PTCA guide-wire assistant in the dTRA group was significantly higher than that in the TRA group (2.8% vs. 0.2%, *P* < 0.01). Compared with that in the TRA group, the dosage of unfractionated heparin in the dTRA group was lower (*P* < 0.05).

**Table 2:** Procedure characteristics

Characteristic	TRA (*n* = 403)	dTRA (*n* = 398)	*P*
Right side first access, % (*n*)	99.8 (402/403)	99.2 (395/398)	0.31
CAG only, % (*n*)	70.2 (283/403)	77.1 (307/398)	0.03
PCI, % (*n*)	29.8 (120/403)	22.9 (91/398)	
Multi-vessel disease, % (*n*)	20.8 (84/403)	13.3 (53/398)	0.08
Number of implanted stents, % (*n*)	26.1 (105/403)	19.8 (79/398)	0.88
Drug balloon, % (*n*)	3.5 (14/403)	3.8 (15/398)	0.82
IVUS, % (*n*)	2.2 (9/403)	2.3 (9/398)	0.98
FFR, % (*n*)	0.2 (1/403)	0.0 (0/398)	1.00
CAD, % (*n*)	54.6 (220/403)	50.8 (202/398)	0.28
ACS, % (*n*)	23.1 (93/403)	18.3 (73/398)	0.10
Sheath size			
6Fr, % (*n*)	99.0 (399/403)	100.0 (398/398)	0.12
7Fr, % (*n*)	1.0 (4/403)	0.0 (0/398)	
PTCA wire assistant, % (*n*)	0.2 (1/403)	2.8 (11/398)	< 0.01
AF radiofrequency ablation, % (*n*)	6.5 (26/403)	5.5 (22/398)	0.58
Dosage of unfractionated heparin, U	3000 (3000–4880) (403)	3000 (3000–3000) (398)	0.03
Crossover site, % (*n*)	2.2 (9/403)	4.5 (18/398)	0.11
ipsilateral radial artery, % (*n*)	0.0 (0/403)	3.3 (13/398)	
ipsilateral distal radial artery, % (*n*)	1.2 (5/403)	0.0 (0/398)	
contralateral radial artery, % (*n*)	0.5 (2/403)	0.8 (3/398)	
contralateral distal radial artery, % (*n*)	0.5 (2/403)	0.5 (2/398)	
femoral artery, % (*n*)	0.0 (0/403)	0.0 (0/398)	

Values are % (*n*/*N*) or median (IQR) (*n*)

*TRA* Transradial access, *dTRA* distal transradial access, *CAG* Coronary angiography, *PCI* Percutaneous coronary intervention, *IVUS* Intravenous ultrasound, *FFR* Fractional flow reserve, *CAD* Coronary artery disease, *ACS* Acute coronary syndrome, *PTCA* Percutaneous transluminal coronary angioplasty, *AF* Atrial fibrillation

Although the overall rate of vascular approach crossover in the dTRA group was high, no significant difference was found between the two groups (4.5% vs. 2.2%, *P* = 0.07). In the TRA group, crossover was required for nine patients. Among them, there were 6 cases of puncture failure, five patients required crossover to the ipsilateral dTRA and one required crossover to the contralateral left dTRA. Although there were 3 cases of successful puncture, there were two cases of radial artery spasm and one case of serious subclavian artery distortion, and conversion to the contralateral vessels was required. In the dTRA group, crossover was required for 18 patients. The puncture site had to be switched for 16 patients due to puncture failure, for 1 patient due to radial artery spasm and for 1 patient due to serious subclavian artery distortion.

### Primary endpoint

Long-term follow-up via ultrasound was obtained in 90.6% of patients in the TRA group and 90.7% of patients in the dTRA group. The characteristics of the patients who were loss-to or completed follow-up are compared in Additional file 1: Table S1.

**Fig. 3:**
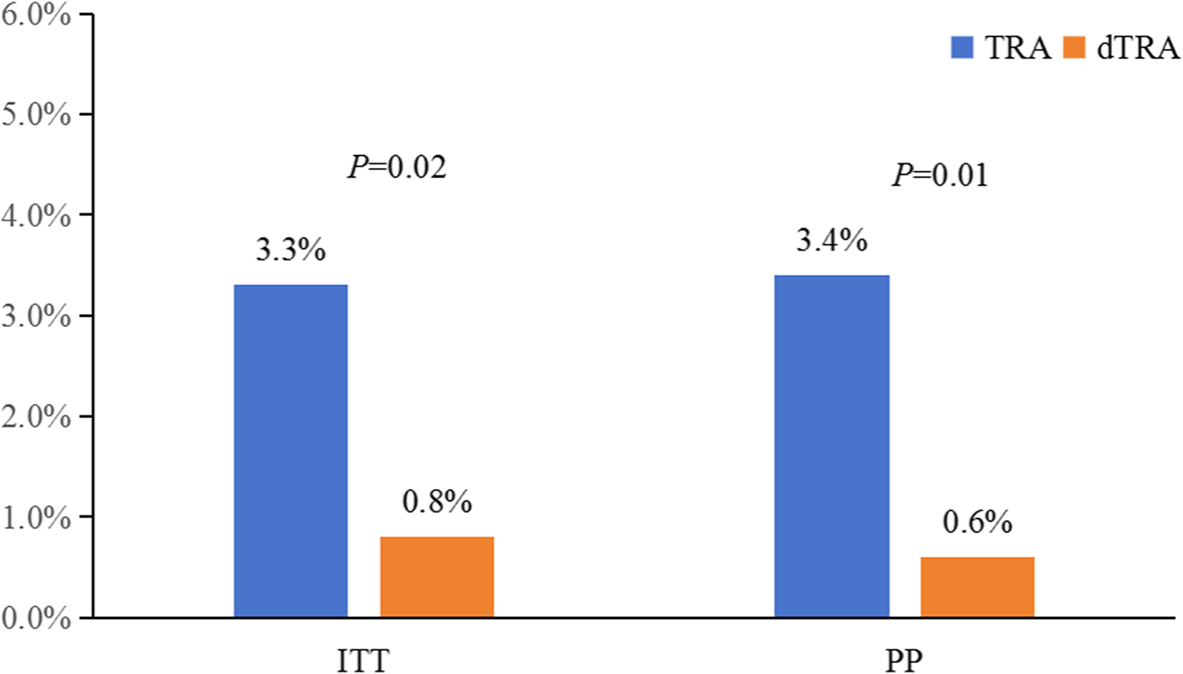
The incidence of long-term RAO

In the ITT analysis, the incidence of RAO at 3 months was 0.8% (3/361) in the dTRA group and 3.3% (12/365) in the TRA group (RR = 0.25, 95% CI = 0.07–0.88, *P* = 0.02). In the PP analysis, the incidence of RAO was 0.6% (2/344) in the dTRA group and 3.4% (12/357) in the TRA group (RR = 0.17, 95% CI = 0.04–0.76, *P* = 0.01), which was consistent with the results of the ITT analysis (Table 3, Fig. 3).

**Table 3:** Primary and secondary outcomes

	**Intention-to-treat**			**Per Protocol**		
	**TRA (*n* = 403)**	**dTRA (*n* = 398)**	***P***	**TRA (*n* = 394)**	**dTRA (*n* = 380)**	***P***
**Primary outcome**						
RAO at 3 months, % (*n*)	3.3 (12/365)	0.8 (3/361)	0.02	3.4 (12/357)	0.6 (2/344)	0.01
**Secondary outcomes**						
RAO at 24 h, % (*n*)	6.7 (27/403)	2.5 (10/398)	< 0.01	5.8 (23/394)	1.6 (6/380)	< 0.01
dRAO at 3 months % (*n*)	0.0 (0/365)	1.4 (5/361)	0.03	0.0 (0/357)	1.2 (4/344)	0.04
dRAO at 24 h, % (n)	0.2 (1/403)	2.3 (9/398)	0.01	0.0 (0/394)	2.4 (9/380)	< 0.01
Success rate of puncture, % (*n*)	98.5 (397/403)	96.0 (382/398)	0.03	100.0 (394/394)	100.0 (380/380)	1.00
Success rate of procedure, % (*n*)	97.8 (394/403)	95.5 (380/398)	0.08	100.0 (394/394)	100.0 (380/380)	1.00
Success rate in single puncture attempt, % (*n*)	83.9 (338/403)	70.9 (282/398)	< 0.01	85.3 (336/394)	73.7 (280/380)	< 0.01
Puncture attempts	1 (1–1) (397)	1 (1–2) (382)	< 0.01	1 (1–1) (394)	1 (1–2) (380)	< 0.01
Puncture time, s	60 (50–60) (397)	60 (50–90) (382)	< 0.01	60 (50–60) (394)	60 (50–90) (380)	< 0.01
Total procedural time, min	30.0 (15.0–47.0) (403)	25.0 (15.0–38.5) (398)	0.06	30.0 (15.0–45.5) (394)	23.0 (15.0–35.0) (380)	0.02
Total fluoroscopy time, min	4.2 (1.6–12.2) (341)	3.7 (1.8–11.0) (324)	0.55	4.2 (1.6–11.9) (332)	3.6 (1.9–10.0) (307)	0.41
Radiation dose, mGy	106.6 (41.5–383.0) (340)	95.5 (39.9–318.5) (324)	0.29	106.2 (41.7–367.9) (331)	93.9 (40.3–290.5) (307)	0.24
Contrast volume, ml	50.0 (50.0–110.0) (403)	50.0 (50.0–90.0) (398)	0.02	50 (50–110) (394)	50 (50–80) (380)	0.02
Bleeding, % (*n*)	6.0 (24/403)	1.5 (6/398)	< 0.01	6.1 (24/394)	1.6 (6/380)	< 0.01
BARC type 1	2.7 (11/403)	0.8 (3/398)	0.03	2.8 (11/394)	0.8 (3/380)	0.04
BARC type ≥ 2	3.2 (13/403)	0.8 (3/398)	0.01	3.3 (13/394)	0.8 (3/380)	0.01
Haematoma, % (*n*)	4.5 (18/403)	4.5 (18/398)	0.97	4.1 (16/394)	3.7 (14/380)	0.79
mEASY type I	2.2 (9/403)	3.8 (15/398)	0.20	2.0 (8/394)	3.4 (13/380)	0.23
mEASY type ≥ II	2.2 (9/403)	0.8 (3/398)	0.09	2.0 (8/394)	0.3 (1/380)	0.04
Finger numbness, % (*n*)	9.2 (37/403)	20.4 (81/398)	< 0.01	9.4 (37/394)	20.3 (77/380)	0.00
Pseudoaneurysm, % (*n*)	0.2 (1/403)	0.0 (0/398)	1.00	0.0 (0/394)	0.0 (0/380)	1.00
Arteriovenous fistula, % (*n*)	0.0 (0/403)	0.0 (0/398)	1.00	0.0 (0/394)	0.0 (0/380)	1.00
Pain during haemostasis	0 (0–1) (395)	0 (0–1) (395)	0.42	0 (0–1) (386)	0 (0–1) (373)	0.46
Change of the thickness of radial artery intima, mm	0.01 (− 0.01–0.04) (403)	0.01 (− 0.01–0.04) (398)	0.72	0.01 (− 0.01–0.04) (394)	0.01 (− 0.01–0.04) (380)	0.72

Values are % (*n*/*N*) or median (IQR) (*n*)

*TRA* Transradial access, *dTRA* distal transradial access, *RAO* Radial artery occlusion, *BARC* Bleeding academic research consortium, *mEASY* modified Early Discharge After Transradial Stenting of Coronary Arteries Study

Post Hoc analysis of the primary outcome was performed by subgroup analysis. In patients with BMI < 24.0 kg/m^2^, the primary outcome occurred in 10 (5.0%) of 200 patients in the TRA group and 1 (0.5%) of 191 patients in the dTRA group (*P* = 0.007). In patients with CAG only, the primary outcome occurred in 11 (3.9%) of 284 patients in the TRA group and 2 (0.7%) of 306 patients in the dTRA group (*P* = 0.008) (Additional file 1: Table S2).

### Secondary endpoints

In the ITT analysis, the incidence of early RAO in the dTRA group was significantly lower than that in the TRA group at 24 h after the procedure (2.5% vs. 6.7%, *P* < 0.01). However, the incidence of dRAO was higher in the dTRA group than in the TRA group in both the early (dTRA: 2.3% vs. TRA: 0.2%, *P* = 0.01) and long-term (dTRA: 1.4% vs. TRA: 0.0%, *P* = 0.03) follow-up (Table 3).

The success rate of puncture (96.0% vs. 98.5%, *P* = 0.03) and single puncture attempt (70.9% vs. 83.9%,* P* < 0.001) was significantly lower in the dTRA group than in the TRA group. However, the number of puncture attempts and puncture time were higher in the dTRA group. There was no difference in the procedure success rate, total procedural time, or total fluoroscopy time between the two groups. Compared with the TRA group, the dTRA group had a larger contrast volume (*P* < 0.05).

The dTRA group had a lower incidence of bleeding than the TRA group (1.5% vs. 6.0%, *P* < 0.01). Additionally, the incidence of hand numbness was significantly higher in the dTRA group than in the TRA group (20.4% vs. 9.2%, *P* < 0.01). There were no significant differences between the two groups in terms of haematoma, pseudoaneurysm, arteriovenous fistula and pain during haemostasis. Compared with that preoperatively, the intima of the radial artery at the 3-month follow-up was significantly increased in both the dTRA group and in the TRA group. However, there was no significant difference in the change in the radial artery intima between the two groups.

In the PP analysis, most of the findings were consistent with the ITT analysis, except for the total procedural time (dTRA: 23 min vs. TRA: 30 min, *P* = 0.02) and the incidence of mEASY type ≥ II haematoma (dTRA: 0.3% vs. TRA: 2.0%, *P* = 0.04).

The results of the comparison of total procedural time and the incidence of mEASY type ≥ II haematoma between the two groups in the as-treated analysis were consistent with those of the PP analysis (Additional file 1: Table S3).

## Discussion

To our knowledge, the present study was the first large-scale randomized controlled study to evaluate the effect of dTRA on the incidence of long-term RAO. The results concluded that dTRA could significantly reduce the incidences of long-term RAO, bleeding and mEASY type ≥ II haematoma, although the rate of successful puncture in a single attempt was lower.

Since Professor Kiemeneij F reported the experience in coronary intervention via the left dTRA, several randomized controlled studies have compared the safety and efficacy of cardiovascular intervention via the dTRA and the TRA, including the incidence of RAO [1, 10, 18–21]. Although most RAO is asymptomatic, an occluded radial artery limits the further use of the radial artery for the repeated catheterization approach and the conduits in patients undergoing coronary artery bypass graft and radial arteriovenous fistula in renal insufficiency in the future. Therefore, preventing and managing RAO is very important [22]. However, the incidences of RAO accrossing studies were inconsistent. In addition, most randomized studies evaluated the incidence of RAO at 24 h after the procedure or before discharge [18, 23–25]. The pooled result in a meta-analysis revealed that the dTRA could reduce the risk of in-hospital RAO (RR: 0.32, 95% CI: 0.19–0.53, *P* < 0.001) [26]. The DISCO trial concluded that the incidence of forearm RAO was low, and was not different between two groups (TRA 0.91% vs dTRA 0.31%; *P* = 0.29) at discharge. However, it needs to be emphasized that systematic implementation of best practices was used to reduce the incidence of RAO in the study, such as reduction of the sheath’s outer diameter, adequate procedural anticoagulation, nonocclusive haemostasis, and a minimal pressure strategy with short haemostasis time. These measures may contribute to the decline in the rate of RAO. However, the incidence of RAO in TRA and dTRA was reported 8.4% *vs.* 0.71% in the DAPRAO trial, and 7.9% *vs.* 3.7% in the ANGIE trial, which was significantly higher than that in the DISCO trial. In our study, we did not take special measures to prevent RAO and found the forearm RAO rate was 6.7% in TRA and 2.5% in dTRA at 24 h post-procedure.

Studies have found that spontaneous recanalization might occur in approximately 30% of patients with RAO in late follow-up [7, 27]. In fact, the long-term outcome of RAO can better reflect the value of the dTRA in the prevention of RAO. To date, there are few randomized controlled studies comparing the incidence of late RAO between the TRA and the dTRA, and the sample size is relatively small, except for the sample in the ANGIE trial [19, 20]. The DAPRAO trial included 282 cases to evaluate the superiority of dTRA for the prevention of RAO and concluded that dTRA can reduce the incidence of RAO at both 24 h and 30 days after a coronary procedure [20]. However, the sample size was relatively small, and only patients with RAO at 24 h were reevaluated after 30 days. As the largest randomized controlled study evaluating the incidence of late RAO, the ANGIE trial included 1042 cases that were followed for a median of 46 days [19]. They found that the incidence of forearm RAO was significantly lower in the dTRA group than in the TRA group (3.7% vs. 7.9%, *P* = 0.01). However, in this study, 15.9% of patients had previously undergone a right dTRA or TRA intervention, and 62.7% of cases required the use of a 5 Fr sheath. The rate of successful sheath insertion was only 78.7% in the dTRA group, which was significantly lower than that in the TRA group. In addition, early RAO before discharge was not reported in the study, and the rate of loss to follow-up was relatively high (23.6%). Repeated ipsilateral radial artery access might aggravate damage to the radial intima and increase the thickness of the intima, which might decrease the successful puncture rate and increase the incidence of access-related complications [28]. In addition, a 6 Fr sheath was indiscriminately used in all patients in our study, which may greatly increase the sheath-to-vessel mismatch in patients undergoing repeated ipsilateral radial artery access. Therefore, we compared the incidence of long-term RAO between dTRA and TRA in patients without a history of ipsilateral radial artery catheterization. In the present study, most patients used 6 Fr sheath, 26.3% of patients underwent coronary intervention, and the incidence of long-term RAO was significantly lower in the dTRA group (ITT: 0.8% vs. 3.3%; PP: 0.6% vs. 3.4%, *P* < 0.05).

Compared with that at 24 h after the procedure, the rate of spontaneous recanalization of RAO at the 3-month follow-up was higher in both the TRA group and the dTRA group (ITT: 50.7% in TRA and 68.0% in dTRA; PP: 41.4% in TRA and 62.5% in dTRA). Previous studies have shown that the rate of recanalization of RAO was approximately 30%; however, in most studies, the follow-up time was approximately 1 month [27]. In the present study, we observed a higher rate of recanalization of RAO and hypothesized that the rate of recanalization might further increase with a longer follow-up time. However, we did not obtain RAO data at the 1-month follow-up, which was one of the limitations of the study. During the follow-up, we also found that patients without early RAO before discharge were prone to no longer occur long-term RAO in either TRA or dTRA.

In the DISCO RADIAL trial, the incidence of dRAO was only 0.46% in the dTRA group after a series of preventive measures for RAO [18]. However, the rate of dRAO was 1.4% in the present study. The distal radial artery was relatively small, and the mismatch between the sheath and the vessel was an independent risk factor for RAO and/or dRAO [29, 30]. To determine whether a slender sheath is superior to a conventional artery sheath in terms of reducing the incidence of dRAO, the ongoing SMART trial (NCT05501925) might provide an answer.

Interestingly, RAO combined with dRAO occurred in 6 patients in the TRA group and in 2 patients in the dTRA group. The occurrence of RAO combined with dRAO in the dTRA is easy to understand. We speculated that the first reason for RAO combined with dRAO might be reverse thrombus formation after RAO. Another reason might be the earlier separation of the superficial palmar arch from the radial artery, which led to the puncture site of the TRA actually being located at the distal radial artery region. Then, the sheath might damage both the conventional radial artery and the distal radial artery.

Although the puncture success rate in the dTRA group was high (96.0%), it was still significantly lower than that in the TRA group (98.5%), and the puncture time was longer. Since 2019, approximately 3000 CAG or PCI procedures via the distal radial artery have been successfully performed in our centre. Main operators have overcome the puncture learning curve and have extensive experience in performing distal radial artery puncture [31].

In recent years, several studies have investigated the impact of the dTRA on the radiation exposure. In the opinion of some scholars, the dTRA should be related to the higher fluoroscopy time and radiation exposure [32]. However, the results were inconsistent. For example, in the ANGIE trial, the total procedure time was longer (14 min vs. 11 min, *P* < 0.001), and the dose area product (DAP) was higher (32,729 vs. 28,909 cGy/cm^2^, *P* = 0.020) in the dTRA group than the TRA group. However, the fluoroscopy time was not significantly different between the dTRA and TRA groups. In a meta-analysis, the authors concluded that the procedure time and the fluoroscopy time were both longer in the dTRA than in the cTRA [33]. Compared with fluoroscopy time, the DAP might be a more comprehensive indicator of radiation exposure. In the present study, there were no significant differences in the total fluoroscopy time or radiation dose between the two groups. Further well-designed studies are needed to confirm whether the dTRA significantly increases radiation exposure.

Regarding other vascular access-related complications, numbness of the hand was common in the dTRA group. The superficial branch of the radial nerve passes through the AS region. Puncture in the AS might damage the superficial branch of the radial nerve, which reminded us to prevent causing a radial nerve injury during the puncture. Compression haemostasis can also cause the numbness of the fingers, but the symptoms usually disappear soon after removing the elastic bandage. However, there were no radial nerve electrograms or ultrasound images to verify this hypothesis in the present study. In addition, although dTRA can reduce the rate of RAO, it might still damage the radial artery and increase the thickness of the intima.

### Study limitations

First, there was no uniform compression haemostasis between the two groups. In the present study, the TR band was used in the TRA group. Due to the lack of special haemostatic devices for dTRA, an elastic bandage was used for haemostasis in the dTRA group. Theoretically, the rate of RAO may be higher with elastic bandages than with compression devices. However, even with the use of elastic bandages for haemostasis, we found a significantly lower rate of RAO in the dTRA group than in the TRA group. That is, the rate of RAO will be lower in the dTRA group with better compression haemostasis. Second, patent haemostasis was not performed in either group, which may have also influenced the outcomes. In “real-world” practice, patent haemostasis is not always feasible in high-volume tertiary centres because it needs more human resources and time. We acknowledge the potential adverse impact without patent haemostasis, but differential bias between groups can be avoided as both TRA and dTRA groups did not use patent haemostasis in our study. Therefore, we think the impact is controllable and relatively acceptable. Third, this study was performed by physicians who were experienced in performing puncture via the TRA and dTRA, which might have increased the puncture success rate and reduced the incidence of radial artery injury. Fourth, according to the previous literature, the initially estimated sample size was 692. In 2022, two large-scale randomized controlled trials with lower difference of incidence of RAO were successively published during our study. We realized that the sample size estimates were overly optimistic in the study design. The statistical power may not be sufficient based on the original estimation protocol. Therefore, we increased the sample size to 801. The incidence of long-term RAO was 3.3% in the TRA group and 0.8% in the dTRA group in the present study as an ITT analysis. Based on the actual outcomes and sample size (801), the post hoc power of the test was only 70%. In principle, we should consider adaptive study of adjusting the sample size when we design the study. Finally, although all patients underwent ultrasound examination before the procedure, this study protocol did not limit the internal diameter of the vessel at the puncture site. As reported, the vessel diameter in AS is significantly smaller than that in conventional radial artery puncture sites, and small vessels may reduce the puncture success rate and increase the risk of RAO [7, 34, 35]. Therefore, preoperative assessment of the internal diameter of the artery by ultrasound and choosing the appropriate patients might further reduce the incidence of RAO.

## Conclusions

In this randomized trial, dTRA significantly reduced the incidence of long-term RAO, bleeding or haematoma, although the puncture success rate was lower.

### Supplementary Information

**Additional file 1: Table S1. **Comparison of the characteristics of patients between loss-to and completed ultrasound follow-up. **Table S2.** Primary outcome in subgroup analysis. **Table S3.** Primary and main secondary outcomes in as-treated analysis.

## Data Availability

The datasets generated and analysed during the current study are not publicly available because of patient privacy but are available from the corresponding author upon reasonable request.

## Abbreviations

AS: Anatomic snuffbox
CAG: Coronary angiography
CI: Confidence intervals
dTRA: Distal transradial access
ITT: Intention-to-treat
PCI: Percutaneous coronary intervention
PP: Per protocol
RAO: Radial artery occlusion
RR: Risk ratios
TRA: Transradial access
