# Supplementary material for: Comparison of long-term radial artery occlusion via distal vs. conventional transradial access (CONDITION): a randomized controlled trial
Source: BMC Med. 2024 Feb 8;22:62. doi: 10.1186/s12916-024-03281-7 (PMC10854098; doi:10.1186/s12916-024-03281-7)
Supplement: Supplementary file 1 — Additional file 1: Table S1. Comparison of the characteristics of patients between loss-to and completed ultrasound follow-up. Table S2. Primary outcome in subgroup analysis. Table S3. Primary and main secondary outcomes in as-treated analysis. [file 12916_2024_3281_MOESM1_ESM.doc]

**Additional file 1**

**Supplementary Tables**

**Table S1. Comparison of the characteristics of patients between loss-to and completed ultrasound follow-up**

| **Characteristic** | **Loss-to follow-up**  **(n=75)** | **Completed follow-up**  **(n=726)** | ***P*** |
| --- | --- | --- | --- |
| **Demographics** |  |  |  |
| Age, years | 70 (64-76) (75) | 66 (57-73) (726) | <0.01 |
| Male, %(n) | 48 (64.0) (75) | 402 (55.37) (726) | 0.15 |
| Body mass index, kg/m^2^ | 24.0 (21.6-26.6) (73) | 24.6 (22.4-26.8) (724) | 0.30 |
| **Medical history** |  |  |  |
| Current smoking, %(n) | 30.7 (23/75) | 29.5 (214/726) | 0.83 |
| Hypertension, %(n) | 61.3 (46/75) | 64.5 (468/726) | 0.59 |
| Diabetes mellitus, %(n) | 17.3 (13/75) | 19.7 (143/727) | 0.62 |
| Dyslipidemia, %(n) | 6.7 (5/75) | 3.4 (25/726) | 0.16 |
| AF, %(n) | 10.7 (8/75) | 13.8 (100/726) | 0.45 |
| Previous PCI with femoral access, %(n) | 1.3 (1/75) | 0.6 (4/726) | 0.41 |
| Previous CABG with LIMA, %(n) | 0.0 (0/75) | 0.6 (4/726) | 1.00 |
| Previous stroke, %(n) | 8.0 (6/75) | 6.2 (45/726) | 0.54 |
| **Echocardiography** |  |  |  |
| LVSD, mm | 31 (29-35) (71) | 31 (29-34) (676) | 0.66 |
| LVED, mm | 48 (46-51) (71) | 48 (45-51) (676) | 0.32 |
| EF, %(n) | 62 (58-67) (71) | 62 (58-66) (676) | 0.56 |
| **Medication** |  |  |  |
| Aspirin, %(n) | 46.7 (35/75) | 43.0 (312/726) | 0.54 |
| Indobufen, %(n) | 1.3 (1/75) | 5.9 (43/726) | 0.10 |
| Clopidogrel, %(n) | 28.0 (21/75) | 32.2 (263/726) | 0.16 |
| Ticagrelor, %(n) | 8.0 (6/75) | 12.1 (88/726) | 0.29 |
| Statin, %(n) | 88.0 (66/75) | 86.4 (627/726) | 0.69 |
| Oral anticoagulation, %(n) | 10.7 (8/75) | 13.8 (100/726) | 0.45 |
| **Diameter of vessels** |  |  |  |
| Radial artery, mm | 2.3 (2.0-2.6) (75) | 2.3 (2.0-2.6) (75) | 0.66 |
| Distal radial artery, mm | 2.0 (1.8-2.2) (75) | 2.0 (1.7-2.3) (75) | 0.99 |
| **Procedure characteristics** |  |  |  |
| TRA , %(n) | 49.3 (37/75) | 49.7 (361/726) | 0.34 |
| CAG only, %(n) | 82.7 (62/75) | 72.7 (528/726) | 0.06 |
| CAD, %(n) | 48.0 (36/75) | 50.7 (386/726) | 0.39 |
| ACS, %(n) | 20.0 (15/75) | 20.8 (151/726) | 0.87 |
| 6Fr Sheath, %(n) | 98.7 (74/75) | 99.6 (723/726) | 0.28 |
| PTCA wire assistant, %(n) | 2.7 (2/75) | 1.3 (10/726) | 0.38 |
| Dosage of unfractionated heparin, U | 3000 (3000-3000) (75) | 3000 (3000-3000) (726) | 0.04 |
| Contrast volume, ml | 50 (50-50) (75) | 50 (50-110) (726) | <0.01 |
| **Endpoints** |  |  |  |
| Successful puncture, %(n) | 98.7 (74/75) | 97.1 (705/726) | 0.43 |
| Successful procedure, %(n) | 97.3 (73/75) | 96.6 (701/726) | 0.72 |
| Success in single puncture attempt, %(n) | 80.0 (60/75) | 77.1 (560/726) | 0.57 |
| Puncture attempts | 1 (1-1) (74) | 1 (1-1) (705) | 0.72 |
| Puncture time, sec | 60 (50-60) (74) | 60 (50-65) (705) | 0.22 |
| Total procedural time, min | 19 (10-30) (75) | 29 (15-45) (726) | <0.01 |
| Total fluoroscopy time, min | 2.5 (1.5-6.1) (63) | 4.2 (1.8-11.8) (602) | 0.03 |
| Bleeding, %(n) | 4.0 (3/75) | 3.7 (27/726) | 0.90 |
| Hematoma, %(n) | 1.3 (1/75) | 4.8 (35/726) | 0.17 |
| Finger numbness, %(n) | 12.0 (9/75) | 15.0 (109/726) | 0.48 |
| Pseudoaneurysm, %(n) | 0.0 (0/75) | 0.1 (1/726) | 0.75 |
| Arteriovenous fistula, %(n) | 0.0 (0/75) | 0.0 (0/726) | 1.00 |
| Pain during hemostasis | 0 (0-0.5) (73) | 0 (0-1) (717) | 0.43 |

Values are % (n/N) or median (IQR) (n).

LIMA, left internal mammary artery; CABG, coronary artery bypass grafting; TRA, transradial artery access; WBC, white blood cell; TC, total cholesterol; TG, triglyceride; LVSD, left ventricular systolic diameter; LVED, left ventricular end-diastolic diameter, EF ejection fraction; APTT, activated partial thromboplastin time; PTCA, percutaneous coronary angioplasty.

**Table S2. Primary outcome in subgroup analysis**

|  | **No./total (%)** | | ***P*** |
| --- | --- | --- | --- |
|  | **TRA (403)** | **dTRA (398)** |  |
| Over all | 12/403 (3.3) | 3/398 (0.8) | 0.020 |
| Age, y |  |  |  |
| < 60 | 4/125 (3.2) | 0/139 (0.0) | 0.049 |
| ≥ 60 | 8/278 (2.9) | 3/259 (1.2) | 0.160 |
| Sex |  |  |  |
| M | 6/227 (2.6) | 1/223 (0.4) | 0.122 |
| F | 6/176 (3.4) | 2/175 (1.1) | 0.283 |
| BMI, kg/m^2^ |  |  |  |
| < 24.0 | 10/200 (5.0) | 1/191 (0.5) | 0.007 |
| ≥ 24.0 | 2/203 (1.0) | 2/207 (1.0) | 1.000 |
| AF |  |  |  |
| yes | 3/62 (4.8) | 1/46 (2.2) | 0.635 |
| no | 9/341 (2.6) | 2/352 (0.6) | 0.029 |
| EH |  |  |  |
| yes | 8/252 (3.2) | 2/262 (0.8) | 0.048 |
| no | 4/151 (2.6) | 1/136 (0.7) | 0.356 |
| DM |  |  |  |
| yes | 3/81 (3.7) | 1/76 (1.3) | 0.621 |
| no | 9/322 (2.8) | 2/322 (0.6) | 0.033 |
| Radial artery diameter, mm |  |  |  |
| < 2.3 | 7/172 (4.1) | 3/174 (1.7) | 0.193 |
| ≥ 2.3 | 5/231 (2.2) | 0/224 (0.0) | 0.061 |
| CAG only |  |  |  |
| yes | 11/284 (3.9) | 2/306 (0.7) | 0.008 |
| no | 1/119 (0.8) | 1/92 (1.1) | 1.000 |

TRA, transradial artery; dTRA, distal transradial artery; CI, confidence interval; M, male; F, female; BMI, body mass index; AF, [atrial fibrillation](https://fanyi.so.com/?src=onebox#atrial%20fibrillation); EH, essential hypertension; DM, diabetic mellitus; CAG, coronary angiography.

**Table S3. Primary and main secondary outcomes in as-treated analysis**

| **Characteristic** | **As-treated analysis** | | |
| --- | --- | --- | --- |
|  | **TRA**  **(n=412)** | **dTRA**  **(n=389)** | ***P*** |
| **Primary outcome** |  |  |  |
| RAO at three months, %(n) | 3.2 (12/374) | 0.9 (3/352) | 0.03 |
| **Secondary outcomes** |  |  |  |
| RAO at 24h, %(n) | 7.0 (29/412) | 2.0 (8/389) | <0.01 |
| dRAO at three months, %(n) | 0.3 (1/374) | 1.1 (4/352) | 0.20 |
| dRAO at 24h, %(n) | 0.0 (0/412) | 2.5 (10/389) | <0.01 |
| Total procedural time, min | 30.0 (15.0-48.0) (412) | 23.0 (15.0-35.5) (389) | 0.01 |
| Bleeding, %(n) | 5.8 (24/412) | 1.5 (6/389) | <0.01 |
| BARC type 1 | 2.7 (11/412) | 0.8 (3/389) | 0.04 |
| BARC type ≥2 | 3.2 (13/412) | 0.8 (3/389) | 0.02 |
| Hematoma, %(n) | 4.9 (20/412) | 4.1 (16/389) | 0.61 |
| mEASY type I | 2.4 (10/412) | 3.6 (14/389) | 0.33 |
| mEASY type ≥II | 2.4 (10/412) | 0.5 (2/389) | 0.03 |
| Finger numbness, %(n) | 10.0 (41/412) | 19.8 (77/389) | <0.01 |

Values are % (n/N) or median (IQR) (n). TRA, transradial access; dTRA, distal transradial access; RAO, radial artery occlusion; BARC, bleeding academic research consortium; mEASY, modified Early Discharge After Transradial Stenting of Coronary Arteries Study.
